# Dermatology Journal Advisory Boards and editorial independence

**DOI:** 10.1016/j.jdin.2023.08.001

**Published:** 2023-08-19

**Authors:** Heidi Bai, Gloria Lin, Jonathan Kantor, Dirk Elston, Jane M. Grant-Kels, Dedee F. Murrell, Jenny E. Murase

**Affiliations:** a University of Rochester School of Medicine & Dentistry, Rochester, New York; b Department of Dermatology, University of Connecticut, Farmington, Connecticut; c Department of Dermatology and Center for Global Health, University of Pennsylvania Perelman School of Medicine, Philadelphia, Pennsylvania; d Department of Dermatology and Dermatologic Surgery, Medical University of South Carolina, Charleston, South Carolina; e Department of Dermatology, St. George Hospital, Sydney, Australia; f Department of Dermatology, University of New South Wales, Sydney, Australia; g Department of Dermatology, University of California, San Francisco, San Francisco, California; h Department of Dermatology, Palo Alto Foundation Medical Group, Mountain View, California

**Keywords:** dermatology, dermatology journal, journal editor, journal governance, society board

## Abstract

**Background:**

Dermatology journals play an essential role in the distribution and promotion of scientific and medical information. Despite this, there are little data on governance structure with respect to its editors, owners, and journal boards that oversee the day-to-day operations for these entities.

**Objective:**

This study aimed to explore the current governance structure of dermatology journals and best practice recommendations.

**Methods:**

The editors-in-chief of the major dermatology journals participated in an online survey of 29 questions to examine general statistics of each journal, open access model, governance structure, and process for editor selection or dismissal.

**Results:**

Of the 52 journal responses, 29 (55.8%) are society-owned journals with 19 (65.5%) primarily governed by a society board, while 18 (34.6%) have an advisory committee or alternative body. Most editor(s)-in-chief (56.9%) serve between 3- and 5-year terms, while 84.6% have the option of at least one renewal. Even though the selection, evaluation, and dismissal processes differed between the journals, generalized best practice recommendations were developed to help improve their overall organization and management.

**Conclusions:**

The oversight structure of dermatology journals varies, and some do not follow current best practice recommendations. Transparency regarding leadership, governance, and due process is needed to maintain editorial independence and integrity.

**Capsule Summary**

• We report existing structures of dermatology journal governance for journals owned by societies, and some do not follow best practice recommendations.
• We provide best practice recommendations for developing an advisory board that maintains editorial independence.

## Introduction

Medical journals play a crucial role in the vetting and dissemination of new information, and readers depend on the editorial board to select articles that are of robust quality and free of undue special interests. Editors-in-chief are responsible for the selection and curation of manuscripts; owners oversee logistical operations, including finances and personnel. Editorial independence is defined by the World Association of Medical Editors (WAME) as full responsibility over the editorial content without interference from journal owners.^1^ Journals are encouraged to construct an independent and diverse advisory committee/board to maintain policy, help resolve conflict, and emphasize transparency while allowing both owners and editors the ability to vet members.^1^^,^^2^ Some journals utilize an ombudsman system, generally an ex-editor-in-chief, to resolve conflict and ensure transparency. The Council of Science Editors (CSE) recommends a contract among editors, societies, journal owners, and publishers with a set length of contract and methods for addressing issues.^3^

As the structure of some dermatology journals do not adhere to the current best practice guidance described by WAME,^1^ International Committee of Medical Journal Editors (ICMJE),^2^ and CSE,^3^ the purpose of this article is to define the importance of developing a journal governance structure, along with policies to preserve editorial independence. This reflects the collective opinion of a panel of international dermatology editors-in-chief and aims for transparency and shared information to improve the success of the journals. We report consistent guidelines for developing an advisory board structure, including the qualifications of individuals and criteria for dismissal, that allow for editorial independence to be preserved and reduce the likelihood that society’s special interests could sway the peer-review process or the content.

Standard best practices are important to ensure the credibility of published articles so that physicians can make appropriate treatment decisions based upon unbiased vetted evidence. To our knowledge, this is the first time any medical editor group has come together to establish a basic understanding of the interplay between editorial boards and advisory boards. We hope to stimulate discussion among other medical specialties to preserve editorial independence across the field of medicine.

## Methods

An online survey (Table I) was sent to the editors-in-chief of 63 dermatology journals with Scopus scores. The survey consisted of 29 questions pertaining to the editor, society, and governance. All editors answered 11 questions and up to 18 additional follow-up questions. While there may be multiple editors for specific journals, only one verified response was included in the analysis, so each journal represents a unique data point. We explored whether a journal’s ownership by a society was associated with specific governance variables. Statistical analyses were performed using Stata 16 for Mac (Stata Corp). The University of Connecticut Institutional Review Board exemption was obtained.

**Table I:** This is the survey tool utilized in the survey of the dermatology journal editors

Survey question	Response type/options
1. Is your journal open access (OA)?	Yes or no
2. If yes to OA, then is it OA gold with fees?	Yes or no
3. If your journal is OA, are you allowed to be involved in discussions around ways to help authors pay the OA fees?	Yes or no
4. How many issues of your journal do you have per year?	[Free text]
5. Is your journal owned by a society?	Yes or no
6. How is (are) the journal chief editor(s) selected for your journal?	[Free text]
7. If you manage a society journal, does the society board provide the primary governance of the journal?	Yes or no
8. Do you have a governance council or alternative body to a society board to provide governance?	Yes or no
9. Please list the name of the governance council or alternative body.	[Free text]
10. If yes to governance council (or alternative body to a society board), how many people sit on the “Governance Council” or equivalent, and how long are their terms?	[Free text]
11. If yes to governance council (or alternative body to a society board), what inclusion criteria are used to determine the members of the governance council?	[Free text]
12. If you manage a society journal, who in the society oversees the journal chief editor(s)?	[Free text]
13. What evaluation process is in place for your chief editors?	[Free text]
14. What are your chief editor’s term limits?	[Free text]
15. Are the chief editor’s terms renewable?	Yes or no
16. What entity would terminate the chief editor(s) if necessary?	[Free text]
17. If you manage a society journal, what mechanisms are used to facilitate communication between the board and the society?	[Free text]
18. If you manage a society journal, are your editors nonvoting board members of the society?	Yes or no
19. If you manage a society journal, how often does the editor present to the board of the society?	[Free text]
20. If your journal was to switch publishers or search for a new publisher,would your chief editor(s) be one of the major player(s) in the RFP process for selecting a new journal publisher?	Yes or no or not sure
21. What is the name of the journal you are affiliated with?	[Free text]
22. If you manage a society journal, what is the name of the society?	[Free text]

*RFP*, Request for proposal.

## Results

Out of the 63 journals surveyed, responses were received from 52 (82.5%) (Table II). For the number of journal issues per year, there were 51 responses. The majority of the journals published 4 to 6 (51%; 26/51) or over 6 issues per year (45.1%; 23/51), while only 3.9% (2/51) published fewer than 4 issues per year (Table III).

**Table II:** Participating journals

Acta Dermatovenerologica Croatica	Journal of American Medical Association Dermatology
American Journal of Dermatopathology	Journal der Deutschen Dermatologischen Gesellschaft
Anais Brasileiros de Dermatologia	Journal of Applied Cosmetology
Annales de Dermatologie et de Vénéréologie	Journal of Cosmetic Dermatology
Archives of Dermatological Research	Journal of Cutaneous and Aesthetic Surgery
Australasian Journal of Dermatology	Journal of Cutaneous Medicine and Surgery
British Journal of Dermatology	Journal of Cutaneous Pathology
Clinical, Cosmetic, and Investigational Dermatology	Journal of Dermatological Science
Clinical and Experimental Dermatology	Journal of Dermatological Treatment
Clinics in Dermatology	Journal of Dermatology
Cutis	Journal of European Academy of Dermatology and Venereology
Dermatitis	Journal of Investigative Dermatology
Dermatologic Clinics	Journal of Psoriasis and Psoriatic arthritis
Dermatologia Cosmética Medica y Quirurgica	Lasers in Surgery and Medicine
Dermatologic Surgery	Leprosy Review
Dermatology	Melanoma Research
Dermatology and Therapy	Nepal Journal of Dermatology, Venereology, and Leprology
Dermatology Online Journal	Pediatric Dermatology
European Journal of Dermatology	Photodermatology, Photoimmunology, and Photomedicine
Indian Journal of Dermatology	Pigment Cell and Melanoma Research
Indian Journal of Dermatology, Venereology, and Leprology	Practical Dermatology
International Journal of Dermatology	Serbian Journal of Dermatology and Venereology
International Journal of Women's Dermatology	Skin Appendage Disorders
Journal of American Academy of Dermatology	Skin Research and Technology
JAAD Case Reports	Skin Health and Disease
JAAD International	SkinMed

**Table III:** Descriptive journal statistics, society board, and advisory board

Number of journals surveyed	63
Number of journal responses	52
Issues published per year (*n* = 51)	
<4	3.9% (2/51)
4-6	51% (26/51)
>6	45.1% (23/51)
Open access model (see Table IV)	40.4% (21/52)
Type of open access model (*n* = 9)	
Gold	44.4% (4/9)
Hybrid	33.3% (3/9)
Diamond	11.1% (1/9)
Ability to participate in discussions to help authors pay for fees in OA models (*n* = 21)	28.6% (6/21)
Society-owned journals	55.8% (29/52)
Society-owned journals primarily governed by a society board (*n* = 29)	65.5% (19/29)
Society-owned journals with editors as nonvoting board members (*n* = 29)	37.9% (11/29)
How often editors report to society (*n* = 28)	
Yearly	32.1% (9/28)
Twice yearly	32.1% (9/28)
Three times yearly	14.3% (4/28)
Quarterly	10.7% (3/28)
Every 2 years	3.6% (1/28)
Monthly	3.6% (1/28)
Bimonthly	3.6% (1/28)
Advisory board or alternative advisory body	
Number of advisory board members	
Lowest	5
Highest	23
Mean	12
Roles of the advisory board (*n* = 8)	
Hire editors	25% (2/8)
Dismiss editors	25% (2/8)
Negotiate publisher contract	25% (2/8)
Editors are mandated to report regularly to an advisory body	37.5% (3/8)
Advisory council that editors can seek advice from	37.5% (3/8)

### Open access

Of the 52 journals, 40.4% (21/52) had an open access (OA) model, and 28.6% (6/21) of those were allowed to take part in discussions to help authors pay for OA fees (Table III). Of the nine who responded regarding their specific model, 44.4% (4/9) are Gold, 33.3% (3/9) are Hybrid, and 11.1% (1/9) are Diamond. While there is a shift toward OA models for new journals, credible models should be maintained to assure quality and appropriate vetting of data.

Best-practice OA models include Gold, Green, Platinum, and Diamond designations (Table IV). The designation of Gold OA indicates that the author only needs to pay the advanced processing charge if the article is accepted for publication. Green OA designation allows for sharing of a subscription-based article as a free online version, typically after a predetermined amount of time, but it may represent a pre-edited version of the final product. The Hybrid model, as the name suggests, involves aspects of both the traditional subscription and OA models with some articles available to the public, while others are only accessible to subscribers. Currently, cost is the main reason preventing this from being the standard model. The Bronze-delayed model is hosted by the publisher’s website on an advanced processing charge basis from the author, and the article can be viewed without a license, but the availability is typically delayed and may only be accessible for a certain amount of time. The Diamond or Platinum model allows free access for both authors and readers through funding by nonprofit foundations, institutions, or the government but permits authors to retain the copyright to the publication.

**Table IV:** Definitions of types of open access

Type of open access (accessed August 22, 2021: http://www.oaacademy.org/types-of-open-access.html)	Definition
Diamond/Platinum	Article immediately available on open access with no fee to readers or authors.
Gold	Author (or their institution or funding agency) pay a publication fee to publish and allow the article to be available free to readers.
Green	Author publishes an article in any journal. Journal allows author to retain noncommercial rights and post their article in a free accessible institutional or specialist online archive or on a website.
Hybrid	One or more articles in a subscription journal can be made available via open access on the internet, while the rest of the content is only available to those who paid for the subscription fee.
Bronze	Articles are made available on websites hosted by the publisher—either immediately or following a delay—but are not formally licensed for reuse.
Black	Publication is not openly licensed, and reuse rights have not been granted but is shared online illegally.

### Society-owned journals

Society-owned journals represent preeminent scientific organizations, including the American Academy of Dermatology and sister societies. By promoting collaboration, encouraging innovation, and focusing on superior standards, these journals consistently support the research of that field.

Of the 52 journal responses, 29 (55.8%) are society-owned with 19 (65.5%) primarily governed by the society board, which serves as the direct governing body. Among those, 37.9% (11/29) have editors who are nonvoting board members. Communication between the journal and society varies. Based on those who responded, 32.1% of editors report to the society annually, 32.1% biannually, 14.3% three times a year, 10.7% quarterly, and the remaining 10.8% report once every 2 years, monthly, or bimonthly (Table III).

Society-owned journals were more likely to involve the editor-in-chief if a change of publisher was contemplated (logistic regression odds ratio of association [OR]: 7.86, 95% CI: 1.87-33.10, *P* = .005). Since some nonsociety-owned journals are publisher-owned, these data may simply reflect industry consolidation rather than differential levels of editor involvement in journal management. OA status was not associated with society ownership (OR: 1.63, 95% CI: 0.53-5.07, *P* = .396). A trend was seen for the number of issues per year, with society-owned journals tending to publish fewer yearly issues (OR: 0.86, 95% CI: 0.73-1.02, *P* = .083).

### Advisory body

Of the 52 journals surveyed, 18 (34.6%) have an alternative “governing” or “advisory” body, which ranged in size from 5 to 23 members with an average of 12. The terms for these individuals range from 2 to 5 years with an average of 3. Of the 8 journals that responded, 25% can hire editors, dismiss editors with or without cause, and negotiate publisher contracts. While 37.5% are mandated to report regularly to the organization’s governing body, only 37.5% state that the advisory council should play an advisory role to the editors when they seek advice (Table III). The selection process to determine the members of the advisory body varies. Requirements include a previous role as editor-in-chief, current editorial involvement, senior editorial board member, society member, set number of publications, active involvement in research, and leadership or expertise in the field and content.

### Editor(s)-in-chief and editors

The editor(s)-in-chief have immense responsibility to the journal and its readership and must be able to process submissions, facilitate the peer-review process, and ultimately determine the conclusion of submissions. Of note, only 29 editors (59.2%) of 49 journals indicated they would play a major role in the publisher selection process if a new one was required (Table V). Of 51 journals, 56.9% of editor(s)-in-chief serve between 3- and 5-year terms, 17.6% unlimited, 5.9% less than 3 years, and 7.8% over 5 years. In addition, 11.8% have undetermined lengths for their tenure, while 3.9% are unsure of the terms. Of note, out of the 52 journals, 84.6% have renewable terms. The selection process can vary but can include appointment or selection through an interview process by the journal owner, publisher, society board, editorial board, or previous editor-in-chief.

**Table V:** Editor(s)-in-chief

Editor(s)-in-chief evaluation process	
Formal evaluation	65.4% (34/52) Personal and/or journal performance review by the society board, publisher, or publication committee.
No formal evaluation	30.8% (16/52)
Unsure	3.8% (2/52)
Editor(s)-in-chief termination process	
Formal termination criteria	82.7% (43/52) The journal owner, society board, publisher, or advisory board can terminate on the grounds of scientific misconduct or an ethical violation.
No formal termination criteria	11.5% (6/52)
Unsure	5.8% (3/52)
Editor(s)-in-chief terms (*n* = 51)	
<3 years	5.9% (1/51)
3-5 years	56.9% (29/51)
>5 years	7.8% (4/51)
Unlimited	17.6% (9/51)
Unsure	3.9% (2/51)
Renewable editor(s)-in-chief terms	84.6% (44/52)
Editor(s)-in-chief have a major role in publisher selection process (*n* = 49)	59.2% (29/49)

Editor(s)-in-chief are typically evaluated based on personal and/or journal performance review by the society board, publisher, or publication committee. Of the 52 journals, 30.8% (16/52) do not have a formal evaluation process, while 3.8% (2/52) were unsure if such procedures existed. Editor(s)-in-chief may be terminated by the journal owner, society board, publisher, or governance council on the grounds of scientific misconduct or an ethical violation. Of the respondents, 11.5% (6/52) of the journals had no defined termination criteria, and 5.8% (3/52) were unsure.

## Discussion

WAME recommends that journals should have an explicit policy describing their governance and relationship to the sponsoring society and that editors-in-chief should report to the highest governing body of the owning organization and not its administrative officers.^1^ In most medical societies, that would be the board of directors. Among the society-owned journals, 37.9% (11/29) have editors who are nonvoting board members, which may help to facilitate communication between the journal and society board. Other means of communication methods like e-mail, telephone calls, regular meetings, and/or quarterly reports may also be helpful to maintain consistent connection. Based on our analysis, communication between the journal and society varies, with most editors reporting to the society yearly or twice yearly.

ICMJE recommends that owners provide editors at the time of their appointment with a contract that clearly states their rights and duties, authority, the general terms of their appointment, and mechanisms for resolving conflict. The editor’s performance may be assessed using mutually agreed-upon measures, including but not necessarily limited to readership, manuscript submissions and handling times, and various journal metrics. Editors should only be dismissed for egregious behavior on the grounds of scientific misconduct or an ethical violation (Table VI). While the majority of the journals surveyed in our assessment had some type of formal evaluation process, there were still 30.8% (16/52) that did not, and 3.8% (2/52) were unsure if such procedures even existed. In addition, 11.5% (6/52) of the journals had no defined termination criteria, and 5.8% (3/52) were unsure, indicating an overall need for more definitive policies regarding assessment and termination criteria to help journals follow best practice recommendations.

**Table VI:** Best practices regarding editor’s role and oversight from WAME, ICMJE, and CSE

	Editor role and contracts	Appointment and dismissal
ICJME	Owners should provide editors at the time of their appointment with a contract that clearly states their rights and duties, authority, the general terms of their appointment, and mechanisms for resolving conflict. The editor’s performance may be assessed using mutually agreed-upon measures, including but not necessarily limited to readership, manuscript submissions and handling times, and various journal metrics. A medical journal should explicitly state its governance and relationship to a journal owner. The ICMJE adopts the World Association of Medical Editors’ definition of editorial freedom, which holds that editors-in-chief have full authority over the entire editorial content of their journal and the timing of publication of that content. Journal owners should not interfere in the evaluation, selection, scheduling, or editing of individual articles either directly or by creating an environment that strongly influences decisions. Editors should base editorial decisions on the validity of the work and its importance to the journal’s readers, not on the commercial implications for the journal, and editors should be free to express critical but responsible views about all aspects of medicine without fear of retribution, even if these views conflict with the commercial goals of the publisher. Editors-in-chief should also have the final say in decisions about which advertisements or sponsored content, including supplements, the journal will and will not carry, and they should have the final say in the use of the journal brand and in overall policy regarding commercial use of journal content. Journals are encouraged to establish an independent and diverse editorial advisory board to help the editor establish and maintain editorial policy. To support editorial decisions and potentially controversial expressions of opinion, owners should ensure that appropriate insurance is obtained in the event of legal action against the editors and should ensure that legal advice is available when necessary. If legal problems arise, the editor should inform their legal adviser and their owner and/or publisher as soon as possible. Editors should defend the confidentiality of authors and peer reviewers (names and reviewer comments) in accordance with ICMJE policy (see Section II C.2.a). Editors should take all reasonable steps to check the facts in journal commentary, including that in news sections and social media postings, and should ensure that staff working for the journal adhere to best journalistic practices including contemporaneous note-taking and seeking a response from all parties when possible before publication. Such practices in support of truth and public interest may be particularly relevant in defense against legal allegations of libel. To secure editorial freedom in practice, the editor should have direct access to the highest level of ownership, not to a delegated manager or administrative officer. Editors and editors’ organizations are obliged to support the concept of editorial freedom and to draw major transgressions of such freedom to the attention of the international medical, academic, and lay communities.	Owners should only dismiss editors for substantial reasons, such as scientific misconduct, disagreement with the long-term editorial direction of the journal, inadequate performance by agreed-upon performance metrics, or inappropriate behavior, that is, incompatible with a position of trust. Appointments and dismissals should be based on evaluations by a panel of independent experts rather than by a small number of executives of the owning organization. This is especially necessary in the case of dismissals because of the high value society places on freedom of speech within science and because it is often the responsibility of editors to challenge the status quo in ways that may conflict with the interests of the journal’s owners.
WAME	The editors-in-chief’s primary responsibilities are to inform and educate readers, with attention to the accuracy and importance of journal articles, and to protect and strengthen the integrity and quality of the journal and its processes. Owners (whether professional associations or for-profit companies) support the core values and policies of their organization and are ultimately responsible for all aspects of publishing the journal, including its staff, budget, and business policies. The relationship between owners and editors-in-chief should be based on mutual respect and trust and recognition of each other’s authority and responsibilities. Conflicts can damage both the intellectual integrity and reputation of the journal and its financial success. The following are guidelines for protecting the responsibility and authority of both editors-in-chief and owners: Editors-in-chief should have full authority over the editorial content of the journal, generally referred to as “editorial independence.” Editorial content includes original research, opinion articles and news reports, both in print or electronic format, and how and when information is published. Owners should not interfere in the evaluation, selection, or editing of individual articles either directly or by creating an environment in which editorial decisions are strongly influenced. Editorial decisions should be based mainly on the validity of the work and its importance to readers, not the policies or commercial success of the owner. Editors should be free to publish critical but responsible views about all aspects of medicine without fear of retribution, even if these views might conflict with the policies or commercial goals of the owner. To maintain this position, editors should seek input from a broad array of advisors such as reviewers, editorial staff, an editorial board, and readers. Editors-in-chief should establish procedures that guard against the influence of commercial, organizational, and personal self-interest on editorial decisions and should make these procedures clear and transparent to all interested parties. They should be compensated for their work on the journal in a manner that does not create a conflict of interest for the manuscripts they consider (see Conflict of Interest Policy Statement). The limits of editorial freedom are difficult to define in the general case. Editors should be receptive to articles representing all legitimate points of view and should be free to publish any responsible positions. However, owners cannot be expected to retain editors who take strong, consistent, one-sided positions against the core values and policies of their parent organization. Editors-in-chief should report to the highest governing body of the owning organization, not its administrative officers. Major decisions regarding the editor’s employment should be made by this body with open discussion and time to hear from all interested parties. Some organizations have found it useful to establish an independent oversight committee to advise them on major decisions regarding their editor and journal. Both owners and editors should have a meaningful role in appointment of members, since both are stake-holders in the committee’s effectiveness. The work of such committees should be transparent and publicly available. Editors should resist any actions that might compromise these principles in their journals, even if it places their own position at risk. If major transgressions do occur, all editors should participate in drawing them to the attention of the international medical, academic, and lay communities.	The conditions of the editors-in-chief's employment, including authority, responsibilities, term of appointment, reporting relationships, and mechanisms for resolving conflict should be explicitly stated in writing and approved by both editor and owner before the editor is appointed. Those conditions bearing on editorial freedom should be shared with readers by publication in the journal or on its website. Owners have the right to hire and fire editors-in-chief, but they should dismiss them only for substantial reasons such as a pattern of bad editorial decisions, disagreement with the long-term editorial direction of the journal, or personal behavior (such as criminal acts) that are incompatible with a position of trust. It may also be appropriate to end the editor’s service if, for whatever reason, owners and editors find they are unable to work together in a spirit of mutual trust and collaboration. Termination of an editor’s appointment should be a deliberate process, involving open discussion at the highest level of the organization, and should not be precipitous, except for egregious wrongdoing.
CSE	Editor responsibilities toward authors • Providing guidelines to authors for preparing and submitting manuscripts. • Providing a clear statement of the journal’s policies on authorship criteria. • Treating all authors with fairness, courtesy, objectivity, honesty, and transparency. • Establishing and defining policies on conflicts of interest for all involved in the publication process, including editors, staff (eg, editorial and sales), authors, and reviewers. • Protecting the confidentiality of every author’s work. • Establishing a system for effective and rapid peer review (see section 2.3). • Making editorial decisions with reasonable speed and communicating them in a clear and constructive manner. • Being vigilant in avoiding the possibility of editors and/or referees delaying a manuscript for suspect reasons • Establishing clear guidelines for authors regarding acceptable practices for sharing experimental materials and information, particularly those required to replicate the research, before and after publication. • Establishing a procedure for reconsidering editorial decisions (see section 2.1.9). • Describing, implementing, and regularly reviewing policies for handling ethical issues and allegations or findings of misconduct by authors and anyone involved in the peer-review process (see sections 2.1.10 and 3.0). • Informing authors of solicited manuscripts that the submission will be evaluated according to the journal’s standard procedures or outlining the decision-making process if it differs from those procedures. • Developing mechanisms, in cooperation with the publisher, to ensure timely publication of accepted manuscripts (see section 2.1.6). • Clearly communicating all other editorial policies and standards. The following are examples of editorial policies and standards that editors may require of submitting authors: • State all sources of funding for research and include this information in the acknowledgment section of the submitted manuscript. • State in the manuscript, if appropriate, that the research protocol employed was approved by the relevant institutional review boards or ethics committees for human (including human cells or tissues) or animal experiments and that all human subjects provided appropriate informed consent. • Describe in the manuscript methods section how cultured cell lines were authenticated. • State in the manuscript, if appropriate, that regulations concerning the use of animals in research, teaching, and testing were adhered to. Governments, institutions, and professional organizations have statements about the use of animals in research. For examples, see the statements from the Federation of American Societies for Experimental Biology,^1^ the Canadian Council on Animal Care,^2^ and, for links to other informational sites, the University of California, San Francisco.^3^ • When race/ethnicity is reported, define who determined race/ethnicity, whether the options were defined by the investigator, and, if so, what they were, and why race/ethnicity is considered important in the study. • List contributors who meet the journal’s criteria for authorship as authors and identify other support (eg, statistical analysis or writers), with the contributor’s approval, in the acknowledgment section. Some journals may require and publish a statement of author contribution for each article. In addition, some journals have a requirement for original research (sometimes called a guarantor policy) that at least one author who had full access to all the data takes responsibility for its integrity and the accuracy of the data analysis. *JAMA* publishes these statements in the acknowledgment section. A description can be found in the *JAMA* Instructions for Authors. • Reveal any potential conflicts of interest of each author either in the cover letter, manuscript, or disclosure form,∗ in accordance with the journal’s policy. • Include (usually written) permission from each individual identified as a source of personal communication or unpublished data. • Describe and provide copies of any similar works in process. • Provide copies of cited manuscripts that are submitted or in press. • Supply supporting manuscript data (eg, actual data that were summarized in the manuscript) to the editor when requested or indicate where (site) the data can be found. • Share data or materials needed by other scientists to replicate the experiment. As an example, the Information for Authors of the *Proceedings of the National Academy of Sciences (PNAS)*† state: “To allow others to replicate and build on work published in *PNAS*, authors must make materials, data, and associated protocols available to readers. Authors must disclose upon submission of the manuscript any restrictions on the availability of materials or information.” • Cite and reference other relevant published work on which the submitted work is based. • Obtain permission from the copyright owner to use/reproduce copyrighted content (eg, figures and tables) in the submitted manuscript, if applicable.‡ • Provide written permission from any potentially identifiable individuals referred to or shown in photographs in the manuscript. • Copyright transfer statement§ or licensing agreement.‖ Some journals may also request or require adherence to the following trial registration or reporting guidelines: • Registration information for clinical trials (See section 2.2.6).¶ • Adherence to the CONSORT statement, which helps standardize reports of randomized trials. • The use of the STARD flow diagram and checklist for reporting diagnostic tests. • Compliance with MOOSE guidelines for reporting meta-analyses and systematic reviews of observational studies. • Compliance with SAGER guidelines or reporting of sex and gender information in study design, data analysis, and results. • Adherence to STROBE checklists for the reporting cohort, case-control, and cross-sectional observational studies. • Adherence to QUOROM guidelines for reporting meta-analyses and systematic reviews of randomized controlled trials. • Adherence to the MIAME standards for reporting microarray experiments. • Adherence to any discipline-specific standards for data sharing and/or open access archiving. A resource that provides information about many of the reporting guidelines is the EQUATOR network.	

*CSE*, Council of Science Editors; *ICMJE,* international committee of medical journal editors; *WAME*, world association of medical editors.

∗ A sample disclosure form can be found at: https://jama.jamanetwork.com/data/ifora-forms/jama/auinst_crit.pdf (Accessed November 11, 2019).

† Proceedings of the National Academy of Sciences *(PNAS)* Information for authors. Available at: http://www.pnas.org/misc/iforc.shtml (Accessed November 11, 2019).

‡ An example of information commonly required for permission to reuse copyrighted material can be found at: http://www.nutrition.org/publications/guidelines-and-policies/permissions/ (Accessed November 11, 2019).

§ A sample copyright transfer agreement is available at: https://aacrjournals.org/sites/default/files/downloads/authors/copyright_form.pdf (Accessed November 11, 2019).

‖ A sample licensing agreement is available at: https://www.springer.com/gp/authors-editors/book-authors-editors/book-authors-helpdesk/rights-permissions-and-licensing/19392 (Accessed November 11, 2019).

¶ Some guidelines for registering clinical trials can be found at http://jama.ama-assn.org/cgi/content/full/292/11/1363 (accessed November 11, 2019).

ICMJE adopts WAME’s definition of editorial freedom; editors-in-chief have full authority over the entire editorial content and the timing of publication of that content without fear of retribution. They state that journal owners should not interfere in the evaluation, selection, scheduling, or editing of individual articles either directly or by creating an environment that strongly influences decisions, and that editors should base editorial decisions on the validity of the work and its importance to the journal’s readers, not on the commercial implications for the journal. Interestingly, only 59% of the 49 journals have a major role in the publisher selection process. Editors should be free to express responsible views about all aspects of medicine without fear of retribution, even if these views conflict with commercial goals. ICMJE notes that editors-in-chief should also have the final say in decisions about which advertisements or sponsored content, including supplements, the journal will and will not carry, and they should have the final say in the use of the journal brand and in overall policy regarding commercial use of journal content. To support editorial decisions and potentially controversial expressions of opinion, owners should ensure that appropriate insurance is obtained in the event of legal action against the editors and that legal advice is available when necessary.^1^^,^^2^

ICMJE encourages the establishment of an independent and diverse advisory board to help the editor establish and maintain editorial policy. Only 18 of the 52 journals that were surveyed though actually have an advisory board, and a minority of the journals (37.5%) is actually mandated to report regularly to the governing body. Out of the 8 journals that responded, 25% can hire editors, dismiss editors with or without cause, and negotiate publisher contracts. This highlights a potentially underutilized resource for the journals and the need to establish clear roles and communication.

The selection process to determine the members of the governance council varies. Several requirements that were highlighted by the respondents include previous role as editor-in-chief, current editorial involvement, senior editorial board member, society member, set number of publications, active involvement in research, and leadership or expertise in the field and content. Some members are appointed or elected by society members or current editor(s)-in-chief. The best practice recommendations proposed for selection are the following: members of any journal advisory committee should (1) have been a senior member of at least one high impact factor medical journal’s editorial board for at least 3 years, (2) have published at least 50 peer-reviewed papers, 3) have no relevant conflicts of interest, and (4) recuse themselves appropriately when any conflicts of interest arise. The CSE recommends that Publication Oversight Committee members have experience in journal management and finances, journal promotion, ranking metrics, and publication ethics.^2^

The role of advisory boards should not be to govern but to provide advice to the editors and act as a firewall between the owners and the journal’s editor(s). This allows for editorial independence to be preserved and reduces the likelihood that society’s special interests could sway the peer-review process or the content. Societies which establish governance councils with authority over editors have created structures that conflict with the guidelines of editorial independence described by WAME, ICMJE, and CSE.^1^, ^2^, ^3^ The structure of some dermatology journals does not adhere to current best practice recommendations. Standard best practices are important to ensure the credibility of published articles so that physicians can make appropriate treatment decisions based upon unbiased vetted evidence.

## Conflicts of interest

None disclosed.
